# Management Strategies for Large Brain Metastases

**DOI:** 10.3389/fonc.2022.827304

**Published:** 2022-02-18

**Authors:** Nehaw Sarmey, Tehila Kaisman-Elbaz, Alireza M. Mohammadi

**Affiliations:** Department of Neurosurgery, Rose Ella Burkhardt Brain Tumor and Neuro-oncology Center, Cleveland Clinic, Cleveland, OH, United States

**Keywords:** stereotactic radiosurgery (SRS) treatment, brain metastases (BM), whole brain radiotherapy (WBRT), large brain metastases, surgery for brain metastases

## Abstract

Brain metastases represent the most common intracranial neoplasm and pose a significant disease burden on the individual and the healthcare system. Although whole brain radiation therapy was historically a first line approach, subsequent research and technological advancements have resulted in a larger armamentarium of strategies for treatment of these patients. While chemotherapeutic options remain limited, surgical resection and stereotactic radiosurgery, as well as their combination therapies, have shifted the paradigms for managing intracranial metastatic disease. Ultimately, no single treatment is shown to be consistently effective across patient groups in terms of overall survival, local and distant control, neurocognitive function, and performance status. However, close consideration of patient and tumor characteristics may help delineate more favorable treatment strategies for individual patients. Here the authors present a review of the recent literature surrounding surgery, whole brain radiation therapy, stereotactic radiosurgery, and combination approaches.

## Introduction

Brain metastases occur in up to 30% of systemic cancers and represent the most common type of intracranial tumor, with significant burden on patient survival and quality of life (1–3). Their management, however, remains complex and controversial. Multiple treatment modalities have been investigated, including surgical resection, radiotherapy (RT), stereotactic radiosurgery (SRS), and chemotherapy (3). Furthermore, large brain metastases, typically defined as ≥ 2 cm in maximum diameter or ≥ 4 cm3 in volume, present additional challenges in management due to their morphology, dosimetry, and anatomy that may be involved. While various chemotherapeutic mechanisms have yielded limited efficacy in the intracranial environment, both surgery and radiation are demonstrated to be promising approaches in this patient population.

The randomized, prospective trial described by Patchell et al. in 1990 remains pivotal in our understanding of the role of the neurosurgeon in the context of whole brain radiation therapy (WBRT) (4). In this study 48 patients were randomized to either surgical resection of their brain tumor followed by WBRT (surgical arm) or needle biopsy followed by WBRT (radiation arm). In the surgical arm, local recurrence was found to be reduced (20% vs. 52%), and overall survival was significantly improved (median 40 weeks vs. 15 weeks in the radiation arm). Additionally, surgically-treated patients retained functional independence over a longer period (38 weeks vs. 8 weeks in the radiation arm). The benefits of surgery were similarly shown by Vecht et al. in 1993 (5). A prospectively randomized trial was conducted in 63 patients with solitary brain metastases, and the addition of surgery to WBRT resulted in significant longer survival and functional independence. These differences were especially notable when stratified according to stable extracranial disease (versus progressive). The utility of surgical resection, therefore, makes it an attractive modality for these patients.

Nonetheless, surgery remains one cornerstone in our paradigm for brain metastases management. The advancement of radiotherapies, including SRS, and our deepening understanding of patient and disease factors have revealed a multi-modal nature of brain metastases management. Here we provide an overview of the role of both surgical and radiation strategies for treatment of large brain metastases, as well as the implication for management of different patients.

## Overview Of Radiation And SRS

Radiation therapy has been a key player in the treatment and palliation for brain metastases, and the technologies and techniques utilized have evolved over many decades (3). Chao et al. first described WBRT in brain metastases patients in 1954, and others have since reported on various outcomes following WBRT (6). As a non-invasive strategy, WBRT is shown to produce a median survival of 4 to 6 months and excellent improvement in ≥70% patients in terms of overall symptoms (7–9). WBRT regimens may also be tailored to the patient (e.g. 20-40 Gy over 1-4 weeks). Various fractionation schedules are utilized, and studies through the Radiation Therapy Oncology Group (RTOG) have revealed the importance of individual patient characteristics in guiding these treatment parameters (10, 11). Both short-term adverse effects, such as fatigue and reversible hair loss, as well as long-term effects, such as cognitive decline and urinary incontinence may influence the decision-making process between patient and physician (12). Some prior studies haves shown significant concern for neuro-cognitive decline within 5 to 36 months, including evidence of white matter changes and cortical atrophy; however, others have suggested that these long-term consequences may be irrelevant when looking at modern-day lower fractionation schemes (<3Gy per fraction) and that the risks of recurrent disease may in fact outweigh these side effects (12–14). In the setting of large brain metastases, WBRT appears to have limited efficacy as shown by Nieder et al. (15) Among 108 patients with 336 brain metastases, local failure was 48% in tumors <0.5cc while all lesions >10cc recurred. Complete response was only seen in tumors <6.4cc (16).

The advent of SRS systems has provided new options for patients in the context of radiation therapy, and its efficacy is supported across high-quality studies (2, 17–19). SRS utilizes multiple non-coplanar beams to deliver single or multi-fraction, highly concentrated radiation doses to a small, precise target volume. This results in a peak dose applied to the central portion of the tumor region of interest, with a steep fall-off gradient out to the periphery. SRS is an interdisciplinary treatment process involving typically a neurosurgeon, radiation oncologist, and radiation physicist to determine the optimal delivery plan.

SRS can be further divided into separate modalities based on the technological systems used, including multiple cobalt-60 sources (Gamma Knife or GK) or single-source linear accelerator (LINAC) (20). GK was initially developed and described by Lars Leksell in 1951, and this utilizes a stereotactic head frame. This tends to offer high conformality to irregularly shaped tumors and the ability to target multiple tumors in the same session. LINAC was developed later in the 1980s and utilizes a collimated, high-energy x-ray beam. Here the LINAC gantry is rotated around the region of interest to produce “multiple noncoplanar intersecting arcs of radiation” (21). Park et al. recently reviewed trends in SRS based on adult patients with non-small cell lung cancer using the National Cancer Database (21). Out of 1780 patients, 77% received GK and 23% underwent LINAC across the study time frame. The usage of LINAC increased steadily from 3.2% in 2003 to 30.8% in 2011 and appeared to be used more widely in community settings, possibly due to lower costs, easier use, less stringent federal regulations, and applicability of some LINAC systems to extra-cranial pathologies. Furthermore, volumetric modulated arc therapy (VMAT) is a more recent modification in LINAC systems, allowing for treatment of multiple targets *via* a single isocenter (single-isocenter multi-target,or SIMT) and reducing overall treatment time.

In a multi-institution series reported by Wen et al. the application of SRS for treatment of brain metastases showed excellent local control (65-90%) and survival (6-12 months). Doses of 15-30Gy were utilized in these patients with acceptable side effect profiles (17). In fact, the maximum tolerated radiation dose for single-fraction radiosurgery has been described as a function of tumor size in order to optimize treatment strength with toxicity profile. Shaw et al. reviewed 156 patients with recurrent primary brain tumors or brain metastases which were previously irradiated (22). They identified maximum tolerated doses (measure at the tumor margin) of 24 Gy, 18 Gy, and 15 Gy for tumors <2 cm, 2.1-3cm, and 3.1-4 cm in maximum diameter, respectively. Thus, larger tumors are typically subjected to lower radiation doses to mitigate toxicities.

Given such dose protocols established through RTOG 90-05, several studies have evaluated local recurrence rates. Vogelbaum et al. assessed 202 patients with 375 brain metastases in a single-center retrospective study after treatment with SRS (23). A dose of 24 Gy to the tumor margin had a significantly lower risk of local failure than 15 or 18 Gy (p = 0.0005), while the 15- and 18-Gy groups were not significantly different from each other (p = 0.82). At 1 year post-SRS, the local control rate was 85% (95% CI 78-92%) in the 24 Gy group, compared with 49% (CI 30-68%) in the 18 Gy group and 45% (CI 23-67%) in the 15 Gy group. Interestingly, overall survival was shown to be unrelated to tumor margin dose. Similarly Petrovich et al. showed that 1-year local control of lesions <3cc was improved compared to lesions >3cc (90% vs 78%), and Ebner et al. showed that large brain metastases with diameter at least 3cm had poorer 1-year local control (68%) compared to smaller lesions (86%, p<0.001) (16, 24, 25).

Tumor size and consequently, radiation dose, also carries an impact on adverse effects. Specifically, radiation necrosis limits the deliverable dose and can have severe neurological impact requiring additional treatments such as steroids and anti-angiogenic drugs. Miller et al. evaluated 5747 brain metastatic lesions in 1939 patients to identify rates of radiation necrosis (26). After SRS treatment at a single tertiary-care center, it was shown that 427 lesions (7%) in 285 patients (15%) developed radiation necrosis at a median of 7.6 months. In multivariate analysis, the lesion diameter (HR 1.29; CI 1.20-1.39) as well as other biological characteristics were independent predictors of radiation necrosis in this population. This included graded prognostic assessment, renal pathology, and heterogeneity index. Certain subsets of pathologies such as HER2-amplified status, BRAF V600+ mutational status, lung adenocarcinoma histology, and ALK rearrangement were also associated with RN.

With respect to RN seen in specific SRS systems, Sebastian et al. recently described a multi-institutional experience including 391 patients treated for 2699 lesions (1014 LINAC-SIMT and 1685 GK) (27). GK was associated with similar overall survival compared to LINAC (9.5 vs 13.2 months), and after propensity score matching using a subset of 113 matched pairs, there remained no significant difference in survival (HR=0.86, p = 0.41). GK meanwhile was associated with higher rate of RN (HR=3.83, p = 0.002) compared to LINAC. Navarria et al. in 2018 presented a randomized clinical trial comparing GK (80 patients) with a LINAC-based Edge SRS system (88 patients) (28). For GK, a single dose of 20-24 Gy at the 50% isodose line was prescribed, whereas for LINAC a single dose of 24 Gy was prescribed; up to four brain metastases with maximum tumor diameter of 3cm were treated per patient. There was no significant difference in overall survival and local control rates between treatment arms. RN was similar except for grade III RN events, which were increased in the GK arm (3 cases at a median time of 3 months; 0 cases in LINAC arms). Thus, while GK remains more commonly used across treatment centers and provides higher dose conformality, data suggests that LINAC systems may have a favorable toxicity profile without negatively impacting survival outcomes.

Whereas SRS may be limited with certain tumor features and carries a risk for radiation necrosis, it offers key advantages compared to surgical resection (29–31). SRS is a less invasive intervention, has shorter procedural times and hospital length of stay, and has less risk of tumor seeding. Surgery alone allows for more immediate improvement of mass effect, formal tissue diagnosis, and no risk of radiation necrosis. The literature has shown the merits of both surgery and SRS in brain metastases patients. Bindal et al. in 1996 compared 13 patients who underwent SRS with 62 patients who underwent surgery, whom they retrospectively matched (32). SRS-treated tumors had a median size of 1.96 cm3 (range 0.41-8.25 cm3) and the median dose was 20 Gy (range 12-22 Gy). Median survival was 7.5 months for patients treated by SRS and 16.4 months for those treated by conventional surgery. Thus, the authors concluded that surgery was a superior option in these patients. Another study by Cho et al. assessed 225 single brain metastases in patients treated with WBRT alone, surgery plus WBRT, or SRS plus WBRT (33). Here the actuarial survival times were similar in the surgery and SRS groups, both of whom responded better than the WBRT alone group. The authors described that SRS may be a more desirable option compared to surgery when lesions are in surgical inaccessible locations and that it is potentially more cost-effective and less invasive to the patient.

Another consideration with SRS is the timing of recurrence compared with modalities such as surgery. Churrilla et al. reported a secondary analysis to compare patients treated with SRS or surgical resection from a phase 3 trial (34). 268 patients with one to three brain metastases were included, of whom 154 underwent SRS and 114 underwent surgery. The surgical arm tended to have larger metastases (median 2.8 cm vs. 2 cm, p<0.001) and more often 1 single brain metastasis (98.2% vs. 74%, p<0.001). Overall local recurrence was found to be similar between treatments (HR 1.15; 95% CI 0.72-1.83). Interestingly, when stratified by time intervals, surgery resulted in a higher risk of early (0-3 months) local recurrence compared with SRS (HR 5.94; 95% CI 1.72-20.45). By 9 months or longer, surgical patients showed a lower risk of local recurrence compared with SRS (HR 0.36; 95% CI 0.14-0.93). Thus, SRS-treated patients showed an advantage in reducing early local recurrences compared to surgery.

## Combination Of SRS and WBRT

With adoption of SRS techniques, clinicians subsequently investigated the role of combination therapy with WBRT. Multiple studies have shown improved local control with this combination approach. Andrews et al. conducted a multi-institutional trial as part of RTOG to compare WBRT against WBRT followed by SRS boost (35). 333 patients with one to three brain metastases were randomly assigned to either treatment arm (167 received WBRT plus SRS; 164 received WBRT alone). Median survival time was significantly higher with combination therapy (6.5 vs 4.9 months, p=0.039). Also, functional status measured by Karnofsky Performance Status (KPS) was more likely to be stable or improved after combination therapy at 6 months (43% vs 27%, p=0.03).

Aoyama and associates described their phase 3 randomized, controlled trial comparing SRS alone with WBRT plus SRS boost in 132 patients (36). Each patient had one to four brain metastases, each less than 3 cm. 65 patients underwent WBRT plus SRS and 67 patients underwent SRS alone. At 1 year, the recurrence rate was significantly lower for combination therapy at 46.8%, compared to 76.4% after SRS alone (p<0.001). More patients required salvage therapy in the SRS group (29 patients versus 10 patients, p<0.001). Median survival was 7.5 months after combination therapy, which was similar to the 8 months survival after SRS alone (p=0.42). Toxicity and death related to neurologic dysfunction were also not shown to be significantly different between the treatment arms.

Kocher et al. reported findings from a phase 3 trial, which evaluated the effect of adding WBRT (30 Gy in 10 fractions) to surgery or SRS (37). Of 359 patients, 199 received SRS (100 patients subsequently observed; 81 subsequently underwent WBRT), and 160 received surgery (79 patients subsequently observed; 81 subsequently underwent WBRT). Here the primary endpoint was deterioration to a WHO performance status (WHO PS) of more than 2. The median time to WHO PS of more than 2 was similar across groups (10 months after observation and 9.5 months after WBRT, p=0.71). Overall survival was also similar at 10.9 months for WBRT and 10.7 months for observations (p=0.89). Of interest in the SRS group, the addition of WBRT resulted in lower 2-year progression rates at 2 years both at initial sites (31% vs. 19%, p = .040) and at new sites (48% vs. 33%, p = .023). Consequently, salvage therapies were also more often utilized.

The addition of WBRT to SRS treatment protocols has shown significant benefit for local control in patients with brain metastases, thus reducing the need for salvage therapies. In some studies, performance status and functional independence have also shown improvement. However, a survival benefit has not been consistently demonstrated.

## Staged SRS Versus Fractionated SRS

Given the limitations of SRS at higher lesion sizes, strategies have emerged to help facilitate more effective application of SRS in brain metastases patients. As described earlier, SRS may be delivered as a stand-alone therapy through a single fraction in a single treatment session. In addition, staged and fractionated SRS schemes have been increasingly utilized depending on patient and tumor characteristics (38). Fractionated SRS (FSRS) involves several daily, consecutive treatments with a smaller dose (e.g. 9 Gy per fraction for 3 days). Staged SRS (SSRS) involves typically two fractions separated by approximately one month, utilizing a higher dose scheme (e.g. 15 Gy per fraction each month). Potentially, these alternative dosing schedules allow for better treatment of larger tumors and/or those too close to critical neural structures (39).

Oermann et al. reported a retrospective review across two centers, involving 214 patients with radiation-naïve brain metastases who received FSRS (39). Patients were given either a single dose or 2-5 fractions (74 patients), and local control was measured. Furthermore, 30 patients had radio-resistant tumors. No difference in local tumor control was found for single-fraction patients when comparing radiosensitive and radioresistant tumors (p=0.69). For the FSRS group, radioresistant tumors failed more frequently compared to radiosensitive (median local control of 14.4 months versus 41.5 months, p=0.001). Thus, radioresistant tumors appeared to respond better to higher dose, single-fraction therapy instead of FSRS dosing. Murai et al. evaluated 54 patients with 102 brain metastases, of which 61 were defined as large (≥2.5cm in maximum diameter) (40). These large brain metastases were treated with 18-30 Gy in three fractions (if ≥2.5 cm to <4 cm diameter) or 21-35 Gy in five fractions (≥4 cm). A dose escalation scheme was applied as long as patients showed no more than grade 2 toxicities. Here, overall survival was 52% and 31% at 6 and 12 months, respectively. For the large brain metastases, local tumor control rates were 77% and 69% at 6 and 12 months, respectively. These higher-dose FSRS schemes were overall well-tolerated and provided good local control and survival in these patients. Navarria et al. described their cohort of 102 patients treated with FSRS (41). They administered 27 Gy in 3 daily fractions to 51 brain metastases measuring 2.1-3cm in diameter; and 32 Gy in 4 fractions was administered for larger tumors measuring 3.1-5cm in diameter. The overall median local control was 30 months with a 1-year local control of 96%. The overall median survival was 14 months with a 1-year survival of 69%. No significant difference was found between the two size groups. Six patients in the cohort developed RN, and all these lesions were larger than 4.1cm in diameter. Overall, large brain metastases showed good response to FSRS.

In another large study of 289 patients with brain metastases >2 cm, Minniti et al. compared single-dose SRS with FSRS (9Gy x 3 days regimen) (42). At one year, local control rates were 77% in the single-dose group compared to 91% in the FSRS group (p=0.01). Radiation necrosis occurred in 31 patients (20%) in the single-dose group compared to 11 (8%) in the FSRS group (p=0.004). On the other hand, Fokas et al. reported their outcomes in a large-scale study of 260 patients treated with single-fraction SRS or FSRS (either 5 Gy × 7 or 4 Gy × 10) (43). Here, no difference was noted in local control at 1 year (73%, 75%, and 71%, respectively; p = 0.191). However, Grades 1–3 toxicity was significantly higher in the SRS group (14%) compared with the FSRS regimens (6% and 2%, respectively; p=0.01). Thus, the lower toxicity profile supported a FSRS scheme in this patient cohort.

Multiple studies have alternatively shown utility of SSRS in certain patient populations with brain metastases. Higuchi et al. evaluated 43 patients with large brain metastases, treated with 30 Gy in 3 staged fractions, delivered over 2 week intervals (44). The local control rates at 6 and 12 months were 89.8% and 75.9%, respectively, and only 1 patient developing a Grade 3 toxicity that required surgery. Of note, tumor volumes decreased by 18.8% (second SSRS) and 39.8% (third SSRS) (p<0.0001). This highlighted the importance of shrinking tumor volumetrics at each subsequent stage in order to achieve better efficacy.

Angelov et al. in 2018 evaluated a 2-stage SRS regimen in 54 patients with 63 large brain metastases (≥2cm) (2). Three primary outcomes were measured: response at first follow-up MRI, time to local progression, and overall patient survival. In this cohort, 46 patients (85%) had a single lesion, 7 patients (13%) had two lesions, and 1 patient had 3 lesions concurrently treated. 14 patients were classified as radioresistant tumors (renal or melanoma). In this staging schedule, the first median dose was 15 Gy (range 12-18) and second was 15 Gy (12-15Gy), in alignment with RTOG 90-05 guidelines. Median duration between stages was 34 days. Ultimately, 9 lesions (14.3%) showed local progression at a median of 5.2 months and 7 (11.1%) showed radiation necrosis (2 confirmed pathologically, 5 assessed based on imaging). Excellent local control at 3 months (95%) and 6 months (88%) was reported. Overall survival rates at 6 and 12 months were 65% ± 7% and 49% ± 8%, respectively. Furthermore, greater tumor volume at baseline was associated with shorter time to progression.

Overall, several retrospective and prospective studies have described individually FSRS and SSRS for management of large brain metastases (2). By increasing dose intensity and spacing out treatments, SSRS may offer improved local control with reduced adverse effects (45, 46). The change in tumor volumetrics at the second or subsequent stages may especially play a role in the overall treatment response. Enhanced tumor cell killing *via* a high dose, followed by an interval period to enable repair of normal cells, may be the mechanism through which SSRS facilitates good local tumor control. Meanwhile FSRS regimens may be a more important option when critical neural structures are involved, thus limiting absolute dosage.

## Surgical Resection Of Brain Metastases

In many patients, surgical resection remains the recommended initial step for treatment of mass effect and brain edema, as well as obtaining a definitive diagnosis. The main surgical techniques, are en-bloc resection which consist of a circumferential resection of the metastatic tumor with tumor capsule preservation, and piecemeal resection. As Patel et al. reported, en-bloc resection demonstrated superiority over piecemeal resection regarding leptomeningeal spread and local recurrence, except for significantly large tumors ≥9.7 cm^3^, for which a 2-times local increased recurrence rate was shown, regardless of the resection technique used (47). Notably, surgical resection as a sole treatment option nowadays, is less acceptable treatment choice for brain metastases. Radiation treatment should accompany it, with appreciation of the radiation modality and timing suitable for each patient.

## Combination Of Surgery And WBRT

Literature reports indicated a significant value in irradiating the intracranial space to provide better local and distant control in proximity to the surgical resection. Nonetheless, the decision of which radiation modality to use relies on the patient’s brain disease burden and expected neurocognitive effect following radiation (3). While some earlier studies suggested no clear benefit for adjuvant WBRT, others have shown encouraging data to support adding WBRT following surgical resection (37, 48–51).

Deangelis et al. evaluated 98 patients who underwent craniotomy for brain metastases resection followed by observation (19 patients) or WBRT (79 patients) (50). Adjuvant WBRT was found to significantly increase time to local or distant failure (p=0.034). At 1 year, the recurrence rate was 22% for WBRT-treated patients and 46% for observation. Median survival between groups was not statistically significantly different (20.6 vs. 14.4 months for WBRT and observation, respectively). Smalley et al. reviewed 85 patients who underwent brain metastases resection, and 34 patients went on to receive WBRT while 51 were observed only (51). The WBRT-treated patients demonstrated lower rates of recurrence (21% versus 85%) and also longer median survival (21 months vs. 11.5 months).

The study by Kocher et al., described earlier here, included one arm of surgery followed by observation or WBRT (37). Here, 160 patients underwent complete resection that was determined macroscopically, imagery or by a combination of both, of whom 79 were subsequently observed and 81 underwent adjuvant WBRT. Notably, the operated study arm included solitary large metastases, as these lesions more frequently required surgical resection. The authors noted that WBRT reduced the probability of relapse at initial sites from 59% to 27% (p<0.001) and at new sites from 42% to 23% (p=0.008). Overall survival and performance status were comparable between groups. Thus, WBRT appears to provide benefit especially in terms of local control without significantly enhancing overall survival.

## Combination Of Surgery And SRS

### Surgery Followed by Adjuvant SRS

Given the potential neurocognitive toxicities associated with WBRT, post-operative adjuvant SRS offers another approach to improve local control when additional treatments are needed. Choi et al. retrospectively evaluated 112 patients with 120 surgical cavities, who subsequently underwent SRS (52). At 1 year, the local failure and distant failure rates were 9.5% and 54%, respectively. When a 2-mm margin was added to the surgical cavity for delivery of SRS, the local failure rates improved (3% versus 16%, p=0.042). There was no significant difference in toxicity at 1 year (3% versus 8%, p=0.27). Median overall survival was 17 months, and the 12-month overall survival rate was 62%. Of note, this methodology of applying a 2-mm margin to the treatment plan stems from prior work by Soltys et al. where 72 patients were treated with SRS alone, resulting in a 79% local control rate at 1 year (53). The authors described that increasing conformality indices (i.e. less conformal plans) were associated with improved local control. Hence, a 2-mm margin technique was advocated and has been adopted by many since then.

Mahajan et al. reported a randomized, controlled, single-center, phase 3 trial comparing post-operative SRS versus observation alone (54). 132 patients who underwent complete resection of one to three brain metastases were assigned to either observation (n=68) or SRS (n=64). In the SRS group, a 1-mm margin are added to the treatment plan. Dosage used was 16 Gy (<10cm3), 14 Gy (10.1-15 cm3), or 12 Gy (>15 cm3) based on cavity volume. Median follow-up was 11.1 months, and the 1-year local control was 43% in the observation group and 72% in the SRS group (HR 0.46, p=0·015). There were no adverse events in either group. Hence, the authors concluded that post-operative SRS offers a significant advantage in treatment.

Brown et al. directly evaluated postoperative SRS against WBRT in a randomized, controlled, phase 3 trial (55). In this multi-center study across 48 institutions, patients with one resected brain metastasis and resection cavity less than 5 cm diameter were eligible for enrollment. Overall 194 patients were assigned to either SRS (12-20 Gy single fraction, using 2-mm margin) or WBRT (30 Gy in 10 daily fractions or 37.5 Gy in 15 daily fractions). Median follow-up was 11.1 months. Importantly, the SRS arm showed a lower risk of cognitive deterioration (median 3.7 months, compared to 3 months for WBRT), and at 6 months the SRS patients had significantly lower rates of cognitive decline (52% compared to 85% of WBRT patients). Median survival was not significantly different (12.2 months for SRS; 11.6 months for WBRT). These findings suggest that SRS is associated with improved neurocognitive outcomes over time without reducing overall survival when compared with WBRT.

It is important to note also that radiation dosing is generally de-escalated for SRS and is variable between treatment centers. Interestingly, local tumor control in the study by Brown et al. was worsened following SRS (median time to progression of 6.4 months) compared with WBRT (median 27.5 months, p<0.0001) (55). The 1-year surgical bed control was 60.5% for SRS patients, relatively lower than that reported by Mahajan et al. (54) In an earlier observational study by Jensen et al. in 2011, 112 resection cavities were treated with SRS under different dosing protocols, reporting a median radiosurgical dose of 17 Gy to the tumor margin and a median cavity volume of 8 cc (56). Here median survival was 10.9 months while local tumor control was 80.3% at 1 year. The continued variability in SRS dosing protocols therefore limits direct comparisons across radiation-based studies.

In the course of post-SRS follow-up, multiple studies have suggested a high risk of leptomeningeal disease (LMD) in this patient population (1). Up to 30% of these patients may go on to develop LMD. Prabhu et al. reported a study of 125 patients who underwent surgical resection and adjunctive SRS to 1 brain metastatic lesion (1). Neurologic death (ND) was measured based on neurologic dysfunction attributable to brain metastases or the associated therapy, without systemic decline or progression. Ultimately, there were 107 patients (86%) who went on to receive LMD salvage treatment, and 82 (66%) also had cranial MRI follow up to characterize radiographic patterns of LMD including classical “sugar-coating” and nodular patterns. ND was seen in 99 patients (79%). These incidences of LMD and ND are in fact higher than the 14% to 48% rates reported in the literature for single-modality therapy (e.g. surgery or SRS) (36, 54, 57, 58).

### Neo-Adjuvant SRS Followed by Surgery

The notable risk of LMD and ND after adjuvant SRS has led to the study of neo-adjuvant SRS (NSRS) as an alternative option to improve patient outcomes. NSRS may allow for more precise definition of the target volume and reduce intraoperative seeding of tumor cells. Asher et al. described a cohort of 47 patients (23 database, 24 prospectively accrued) with 51 lesions (59). NSRS was done a median of 1 day before surgical resection. Median lesion diameter was 3.04cm with a mdian volume of 8.49 cc). Median dose was 14 Gy to 80% isodose line. After a median follow-up of 12 months, overall survival was 77.8% and 60% at 6 and 12 months, respectively. Local control rates were 97.8% and 71.8% at 6 and 24 months, respectively. Interestingly, no LMD or other perioperative adverse events were reported. 8% of patients went on to develop radiation necrosis. Local failure was more likely with lesions >3.4 cm, and six of the 8 failures had a dural attachment or proximity to draining veins. Thus, NSRS yielded excellent response rates in this cohort with low rates of radiation necrosis and LMD.

Prabhu et al. in 2017 conducted a retrospective, multi-institutional study of 213 patients to determine outcomes of SRS alone or SRS plus surgery (60). 223 large brain metastases (≥4cm) were treated with either SRS alone (61), NSRS and surgery (62), or surgery with adjuvant SRS (94). Any complete resection with SRS was associated with improved local control (79.5%) compared with SRS alone (63.3%). Postoperative SRS resulted in the highest rate of radiation necrosis (22.6%) compared to SRS alone (12.3%) and NSRS (5%). In a more recent and updated analysis of their NSRS patients, Prabhu et al. in 2018 described 117 patients with 125 lesions treated with NSRS (63). Gross total resection was achieved in 95.2% of lesions, and median SRS dose was 15 Gy. Local recurrence at 2 years was 25.1% and distance failure was 60.2%. LMD was found in 4.3% of cases, and symptomatic radiation necrosis occurred in 4.8% of cases. Median overall survival was 17.2 months. Thus, NSRS resulted in good local control with an acceptable low toxicity profile.

## Intraoperative Radiotherapy

While SRS and WBRT have been extensively evaluated in the last few decades for management of brain metastases, another mode of radiation therapy that is increasingly gaining attention is intraoperative radiotherapy (IORT) (64). IORT involves a single dose of radiation administered at the same time as the surgical biopsy or resection being performed. Three main categories are described: intraoperative electron radiotherapy (IOERT), low-energy X-ray intraoperative radiotherapy (LEX-IORT), and intraoperative high-dose brachytherapy (IOHDR). IOERT has historically been used in extracranial tumors such as breast, pancreas, head and neck, and colorectal cancers. Generally, it requires a cavity with clear line of sight given the structure of applicator tubes. LEX-IORT utilizes a 30- to 50-kV istotropic X-ray source and adapts more conformally to the resection cavity of interest while applying a more steep dose gradient. IOHDR involves a sealed radionuclide source being placed inside the resection cavity itself. This therapy has been used extensively in rectal cancers, soft tissue sarcomas, and head and neck cancers.

While there is limited data regarding intracranial effectiveness and risks with IORT, early studies do suggest potential benefits from this modality. Weil et al. evaluated 23 patients treated with 50 kV LEX-IORT, where 14 Gy was delivered to a 2mm depth from the applicator surface (62). Progression-free survival from time of surgery was 22 months and overall survival was 30 months (1-year local control of 50%). In another study by Cifarelli et al. 54 patients were treated with LEX-IORT, using a median dose of 30 Gy to the applicator surface (65). The 1-year local control was 88%, and overall survival was 73%. LMD occurred in 3% of patients, and RN occurred in 7% of patients. Kahl et al. reported their cohort of 40 patients with 44 resected metastases, who were treated with LEX-IORT using a median dose of 20Gy (66). Median overall survival was 26.4 months (1- year survival of 61.6%), and the local control was 88.6% (1-year local control of 84.3%). They observed a low RN rate of 2.5%. The potential for favorable progression-free and survival outcomes coupled with a low toxicity profile that is demonstrated in these preliminary findings certainly warrants larger, prospective studies on IORT.

## Histologic Considerations In Treatment

Our increased understanding of molecular genetics in tumor pathogenesis has allowed for more detailed diagnostics as well as tailored treatment options for cancer patients. In the context of patients suffering from brain metastases, it is therefore useful to evaluate histologic background in relation to treatment response. Few of the notable histological categories are discussed here.

In non-small-cell lung cancer (NSCLC), brain metastases may arise in 30% of patients in their disease course (61, 67). A unique subset of tumors carry the ALK rearrangement, which make these patients excellent candidates for targeted treatment with ALK-targeted tyrosine kinase inhibitors (TKI), including crizotinib. Nonetheless, brain metastases frequently occur, likely due to poor penetration of the drug across the blood-brain barrier. The role of radiotherapy in enhancing progression and survival in these patients is unclear. Johung et al. reviewed a cohort of 90 patients with ALK-rearranged NSCLC treated with a combination of SRS, WBRT, and TKI therapy (67). The median overall survival after diagnosis of brain metastases was 49.5 months and median intracranial progression-free survival was 11.9 months. Yang et al. reviewed outcomes from a smaller cohort of 34 patients, of which 19 were treated with combined TKI and radiotherapy, resulting in 70% overall survival at 3 years (68). Thomas et al. retrospectively reviewed 52 ALK-positive NSCLC patients and evaluated TKI combined with radiation versus newer CNS-penetrant TKI therapies alone (69). They reported similar time to intracranial progression (18.1 vs 21.8 mos, p=0.65) and time to overall progression (11.4 vs 13.4 months, p=0.98) for both groups. Thus, radiation with SRS or WBRT represents an important treatment option in these patients, but this should be further evaluated in the context of evolving TKI and other targeted therapies.

Melanoma represents another significant primary tumor histology, wherein 10-73% of patients go on to develop brain metastases (70). Median survival in these patients is 6.74 months, and the relatively radioresistant nature of these tumors makes SRS a more viable treatment option compared to WBRT. Goyal et al. reported a systematic review demonstrating favorable outcomes following SRS therapy in melanoma patients, while the addition of WBRT led to detrimental neurocognitive outcomes and no improvement in overall survival (71). Furthermore, half of melanoma patients carry the BRAF protein kinase mutation, and studies have shown favorable response to BRAF inhibitor therapy (BRAFi). Mastorakos et al. reviewed 198 patients in a multicenter retrospective cohort study to evaluate the role of SRS and BRAF mutation status in brain metastasis patients (70). They found that BRAF-mutated patients (45.5% or 90) receiving BRAFi had improved survival overall compared to wild-type BRAF. After receiving SRS in these two groups, median survival was improved in the BRAFi group compared to the wild-type group as well (13 vs 7 months). Furthermore, in terms of radiation timing, BRAFi given after SRS showed improved survival compared to giving it before or during SRS. While the authors concluded that SRS treatment followed by BRAFi may improves survival outcomes, this must also be weighed against the risks of therapy. Notably, BRAFi treatment was associated with a higher risk of intracerebral hemorrhage compared to no BRAFi treatment (10.4% vs 3%, p=0.03).

Small cell lung cancer (SCLC) has historically been excluded from randomized trials given its unique biology (72). SCLC carries a 40-50% risk of metastasis to the brain and is shown to have high radio- and chemo-sensitivity. The rapidly progressive nature of SCLC has led to the prevalent use of WBRT in its management, with prophylactic cranial irradiation (PCI) shown to increase survival when administered earlier in the course of this disease (73, 74). As imaging and clinical surveillance has improved and neurocognitive outcomes have become more relevant, there is renewed interest in SRS for these patients. Rusthoven et al. described a multi-center retrospective study evaluating 710 SCLC patients treated with SRS without prior PCI or WBRT (75). The median overall survival was 8.5 months, and median time to central nervous system progression was 8.1 months. After propensity matching a subset of 187 patients from the SRS cohort with 187 WBRT-treated patients, the overall survival was higher with SRS (median 6.5 months vs 5.2 months, p=0.003) while no difference was seen in progression-free survival (median 4 months for SRS vs 3.8 months for WBRT, p=0.79). Another study by Cifarelli et al. evaluated 293 patients treated with SRS for SCLC brain metastases across 10 centers (72). In this cohort, 79% had received SRS as salvage therapy following WBRT or PCI. At one year in the overall cohort, the local failure, distant brain failure, and overall survival were 31%, 49%, and 28%, respectively. On multivariate analysis, younger age for patients receiving salvage SRS was a significant predictor of overall survival. Robin et al. reviewed outcomes from the National Cancer Database comparing upfront SRS against upfront WBRT with or without SRS (76). After propensity score matching between 193 SRS patients and 1930 WBRT patients, overall survival was shown to be improved in the SRS-alone group (median 10.9 months vs 7.6 months, p<0.001). The encouraging outcomes with SRS warrant prospective trials to further elucidate its role in SCLC management (77).

## Discussion

Brain metastases represent a major healthcare burden, with significant impact on quality of life and survival (3). Survival length, however, is usually dependent on systemic disease control rather than CNS disease, and even though it may not reflect the efficacy of applied oncological treatment as local control do, it is a commonly measured outcome of studies in this field and therefore is extensively reported in this review as well.

While WBRT was initially a mainstay in treatment, its lack of specificity and risk of neurocognitive decline has required us to seek other modalities for therapy. Several alternative paradigms have been increasingly utilized in the last 30 years, including surgical resection; SRS *via* single, fractionated or staged approaches; and a combination of surgery with radiation (see **Table 1**). Early data evaluating IORT as an alternate mechanism for radiation delivery in intracranial disease remains limited yet encouraging. As the state of research evolves, it is imperative for the neurosurgical oncology community to continually update practice guidelines and metrics for evaluation of treatment modalities (78).

**Table 1:** Overview of treatment strategies and their benefits and risks.

Treatment	Benefits	Risks
Surgical resection	• Relief of mass effect • Obtain pathological diagnosis • Improved survival compared to WBRT	• Most invasive • Peri-operative complications: hemorrhage, wound healing
a. Surgery plus WBRT	• Improved local and distant control compared to surgery alone.	• Long-term neurocognitive effects • Longer treatment duration for patient • Survival benefit inconsistent
b. Surgery plus SRS	• Improved local control compared to surgery alone • Reduced neurocognitive risks	• Leptomeningeal disease • Radiation necrosis
SRS	• High dose delivery in a single treatment session • Less invasive than surgery • Possibly lower rates of early local recurrence	• Radiation necrosis • Limited dose delivery with large brain metastases
a. Fractionated SRS	• Lower doses can be applied when close to sensitive neural elements	• Radiation necrosis
b. Staged SRS	• May help with large brain metastases requiring higher dosage overall	• Radiation necrosis
WBRT	• Less invasive • Good local and distant control	• Limited dose and targeting • Neurocognitive decline • Longer treatment duration for patient • More palliative in nature
a. SRS plus WBRT	• Improved progression-free survival • Preservation of functional status and cognitive function overall	• No consistent survival benefit • Not useful when significant brain edema is a concern

The availability of different options, therefore, allows a more tailored approach to each patient. While surgery offers a direct, immediate method for relieving mass effect and brain edema, SRS offers a less invasive approach with good local control and avoidance of peri-operative complications. In those patients for whom surgery would not be well-tolerated and mass effect is not of immediate concern, SRS alone may be a reasonable approach. Due to dose limitations of SRS in the context of large brain metastases and those lesions that are close to critical neural elements, a fractionated or staged approach may be pursued. Here, the goal is to maximize dose intensity in a safe manner to enhance tumor cell kill, while reducing risk of damage to surrounding structures. Staged SRS may also be valuable in radioresistant tumors where higher doses can be delivered. However, in patients who develop radiation necrosis, additional treatments may be needed, including anti-angiogenic agents.

Finally, a combination of surgery and SRS has been recommended in some cases to further improve local tumor control at the resection cavity. As described earlier, for large brain metastases where mass effect is of concern, surgical resection provides immediate symptomatic relief while SRS boost to a 1 to 2-mm margin is shown to enhance local control. Furthermore, adjuvant SRS may be superior to WBRT in terms of better neurocognitive outcomes. Interestingly, newer paradigms are emerging to address the neurocognitive risks associated with traditional WBRT. The use of memantine and hippocampal-sparing WBRT have been described more recently in brain metastases patients. Brown et al. in 2010 presented a phase III randomized trial evaluating 518 patients over a median follow-up of 7.9 months. Cognitive decline was significantly improved after hippocampal-sparing WBRT plus memantine versus WBRT plus memantine (HR 0.74, p=0.02). No significant difference was reported in progression-free survival or overall survival.

Regarding the notable risk of leptomeningeal disease after adjuvant SRS, several studies have argued that neoadjuvant SRS may be a better therapeutic strategy. Notably, radiation dosages varies in literature reports and further validation is needed as lower radiation doses given to large metastases may eventually lead to a higher recurrence rate. This removes the need for radiation to a post-operative cavity margin, thus reducing risk of radiation necrosis. Also, the sterilization of tumor cells pre-operatively appears to reduce the risk of seeding during surgery, thus reducing the risk of leptomeningeal disease as well. En-bloc gross total resection is additionally important in improving patient outcomes in terms of local control and overall survival.

Ultimately, management of brain metastases remains a controversial issue as a single treatment plan may not apply to most patients. It is at the discretion of the treating neurosurgeon, along with radiation oncologist colleagues, to evaluate the benefits and risks of treatment with each patient.

## Author Contributions

NS: Compiled/interpreted resources and primary author for majority of the manuscript. TK: Assisted with manuscript writing/edits. AM: Conception and overall design of project/paper, manuscript editing/revisions, interpretation of resources to include in paper. All authors contributed to the article and approved the submitted version.

## Conflict of Interest

The authors declare that the research was conducted in the absence of any commercial or financial relationships that could be construed as a potential conflict of interest.

The handling editor declared a past co-authorship with one of the authors AM.

## Publisher’s Note

All claims expressed in this article are solely those of the authors and do not necessarily represent those of their affiliated organizations, or those of the publisher, the editors and the reviewers. Any product that may be evaluated in this article, or claim that may be made by its manufacturer, is not guaranteed or endorsed by the publisher.
